# High Incidence of Bronchospastic Response to a Stair Climbing Exercise

**DOI:** 10.7759/cureus.14843

**Published:** 2021-05-04

**Authors:** Tomas Venckunas, Domantas Balsys

**Affiliations:** 1 Institute of Sport Science and Innovations, Lithuanian Sports University, Kaunas, LTU

**Keywords:** high-intensity exercise, airway hyper-responsiveness, exercise-induced asthma

## Abstract

Background

While it is increasingly recognized that exercise-induced bronchoconstriction (EIB) affects a substantial proportion of sport participants, the relation of EIB incidence and severity to the type and intensity of exertion remains under-investigated. The aim of this study was to establish the incidence and severity of EIB during a stair race, a highly demanding all-out effort exercise. We hypothesized that a large proportion of participants would develop EIB to this particular high-intensity competition, and that among the predisposing factors, severity of EIB would depend on the level of exertion.

Methodology

In this study, screening for EIB was conducted during the official competitive race to a 114-m skyscraper held during the late spring in a city center of approximately 0.5 million residents. Healthy active men (n = 26; age = 32.0 ± 7.0 years) volunteering for the study from the field of the race were included. Allergy Questionnaire for Athletes was completed, and responses of expiratory capacity (forced expiratory volume in the first second, FEV1) and blood lactate were measured by portable digital devices.

Results

On average, FEV1 dropped by 10.5 ± 5.6% after the exercise. In 11 (approximately 42.5%) participants, FEV1 drop was >10%, indicative of clinical EIB. While age, anthropometry, training experience, allergy history, baseline FEV1, and post-exercise lactate did not differ in responders versus non-responders, those with FEV1 decrement of >10% were slower in the race.

Conclusions

Due to very high incidence of EIB observed in active men performing a maximal-effort task and negative association of EIB with competitive performance, the condition of these individuals undertaking regular intense exercise deserves more attention.

## Introduction

Exercise-induced bronchoconstriction (EIB) is described as a temporal airway narrowing leading to impaired airway conductance and decreased airflow capacity as a result of exercise [1]. While a substantial proportion of both asthmatic and otherwise healthy individuals are affected by EIB [2], and endurance exercises are considered to induce bronchoconstriction most frequently, the relationship between the incidence and severity of EIB and the type of exercise remains elusive [1,2].

Different forms of endurance training are increasingly advocated by sport coaches to improve performance as well as by physicians to prevent disease. From an individual perspective, it is important not only to consider the positive effects of such training but also foresee the possible side effects it may have, for example, development of EIB and subsequent sequelae as poor exercise adherence, suboptimal training adaptation, and reduced health benefits. Specifically, due to the emerging popularity of high-intensity interval training, one of the endurance training types, its effects on EIB warrants further investigation. Therefore, the aim of the present study was to assess the incidence of EIB among participants of a skyscraper stair run, a highly intense exercise.

## Materials and methods

Participants

Healthy active Caucasian men (n = 26) engaged at recreational to national level in endurance sports (n = 20, mostly running-based) or basketball (n = 6) participated in the competitive skyscraper race and agreed to the additional procedures of the study (i.e., to complete a questionnaire, body mass measurements, and measurements of blood lactate and forced expiratory volume in one second). Participants signed an informed consent form, and the study was approved by the Regional Biomedical Research Ethics Committee.

Organization and measurements

Participants competed in an official skyscraper stair race to the top of a 31-floor (114 m) skyscraper after warm-up. The competitors started the timed trial with at least 30 seconds between each other. The time taken to complete the course was measured via tracking sensors on the bib attached in the front at the chest level. Air temperature and humidity in the staircase was approximately 20°C and 62%, respectively. The event was held in late spring during pollen season.

Within one hour before the race, before they performed individual warm-up, participants completed the Allergy Questionnaire for Athletes (AQUA), an easy to handle and reliable tool for screening allergy in athletes [3]. Additionally, their body weights in competitive gear without shoes was measured, and pre-exercise spirometry was determined using a portable spirometer (Spirobank II CE 0476, MIR SRL, Rome, Italy) while wearing a nose clip. The spirometry started with full inhalation, followed by forced maximal exhalation, and then full inhalation again in a standing position; several attempts were performed until correct expiration and inspiration curves were achieved. After the race, participants rested standing and spirometry was performed within one minute, and then at two minutes, five minutes, and 10 minutes after the race using an identical spirometer following the same procedures, but supervised by another instructor. The highest pre-exercise forced expiratory volume in the first second (FEV1) value was considered baseline expiratory flow capacity. The largest decrement of FEV1 during the 10-minute post-exercise period was used to determine the presence of EIB, defined as an FEV1 decrement of >10%. The blood lactate concentration was measured using a fingertip blood sample taken two minutes and five minutes after the finish with a pocket analyzer (Lactate Pro 2, Arkray, Japan). As lactate concentrations did not differ significantly between the two time points, the mean value was used for analysis. The pre-exercise lactate concentration was not measured due to logistic constraints associated with the competitive nature of the event.

Statistical analysis

Difference of the means was analyzed using Student’s t-test for dependent or independent samples where appropriate, and a p-value of ≤0.05 was considered significant. The analysis was performed using IBM SPSS Statistics version 26 (IBM Corp., Armonk, NY, USA).

## Results

Exercise induced an average 10.5 ± 5.6% (range: 3.2-29.2%) drop in FEV1 (p < 0.001). Even in the group with an FEV1 decrement of <10% (n = 15), the change was significant (p < 0.05), and 11 (42.3%) participants had FEV1 decrement of >10%, four of which above 15%. The FEV1 decrement did not differ at one, two, five, and ten minutes for both groups after the exercise (p > 0.05).

Although age, anthropometry, training experience, responses to AQUA (up to two positive answers in responders and up to three positive answers in non-responders), baseline FEV1, and post-exercise lactate did not differ between the groups. Those with FEV1 decrement of >10% took more time to complete the race (Table 1). Table 1 also lists participant characteristics.

**Table 1:** Characteristics, expiratory function, and performance measures of the participants. Data are presented as mean ± standard deviation. AQUA: Allergy Questionnaire for Athletes; FEV1: forced expiratory volume in the first second Significant p-values are highlighted in bold

	Responders (n = 11) FEV1 drop >10 %	Non-responders (n = 15) FEV1 drop <10 %	P-Value
Age, years	31.9 ± 8.4	32.0 ± 5.3	0.972
Height, cm	180 ± 8	181 ± 5	0.647
Body mass index, kg/m^2^	23.8 ± 2.1	22.7 ± 2.1	0.241
Training experience, years	13.6 ± 9.2	15.5 ± 8.0	0.605
Average (range) AQUA score	0.9 (0–2) (n = 6)	0.8 (0–3) (n = 8)	0.773
Baseline FEV1, L/s	5.02 ± 0.68	4.98 ± 0.35	0.846
FEV1 decrement, %	15.1 ± 5.9	7.1 ± 1.8	0.0001
Performance time, s	282.5 ± 67.9	231.1 ± 35.5	0.022
Post-exercise lactate, mmol/L	16.7 ± 3.0	17.4 ± 2.0	0.773

## Discussion

This study revealed that very intense exercise lasting approximately four minutes induced a substantial reduction in FEV1. In 40% of the active men enrolled in the current study, this decrement in FEV1 was >10%, which is among the highest reported incidence of EIB in healthy, non-elite adult athletic population [4,5]. It has been reported in a small sample of non-athletes that two types of high-intensity interval training sessions did not induce more pronounced EIB compared with moderate-intensity continuous exercise [6-8], which may be due to an exercise intensity that was not all-out in these studies. The results of the current study suggest that EIB may be underappreciated, even among non-asthmatics. Based on our observation, it needs to be explored whether exercise of similar intensities performed in a more challenging environment (e.g., higher or lower temperature) evokes bronchoconstriction in an even larger proportion of participants. The fact that a challenging environment may have such an impact is shown by the higher incidence of bronchoconstriction during high-intensity exercise by trained adolescents when performed in low versus high temperature [9].

Although factors and mechanisms underlying EIB in apparently healthy young active men could not be discerned in the current study, it was not associated with the training history, age, anthropometrics, reported atopic status, or allergies. EIB was also not associated with the level of exertion, as estimated from post-exercise blood lactate concentration. However, those who developed EIB (as judged by the universally accepted FEV1 decrement cut-off value of >10%) were slower in the race. It might be argued that EIB depends on the duration of exercise or the total amount of air ventilated during the task, and thus, extent of airway desiccation and increase in osmolality of the mucous over the epithelial layer of cells, triggering constriction of smooth airway musculature for those who took more time to complete the exercise. However, we believe a more likely explanation is that EIB during exertion impairs alveolar gas exchange and hinders performance via inadequate oxygen supply to the mitochondria of the active muscles. This reasoning is corroborated by the observation that FEV1 decrement within the first minute after exercise did not differ from FEV1 at later time points in both responders and non-responders. In addition, it has been seen that in asthmatics some bronchoconstriction can occur during submaximal exercise [10]. The prolonged post-exercise impairment of the expiratory capacity also allows us to refute respiratory muscle fatigue as a main cause of FEV1 decrement (as respiratory muscle fatigue would most probably be resolved at least partially within 10 minutes of recovery). Therefore, it is apparent that EIB was triggered by this competitive and highly metabolically demanding event in nearly all participants of the race, but to a different degree, which limited exercise capacity in the responders.

Exercise challenge tests have a high specificity for diagnosing EIB [11-13], and tests performed in the field should be the first choice for competitive athletic individuals [14-16], provided the standardization is similar to the laboratory conditions [12], which was clearly the case in the current study. However, the scope of this study, due to suspension of further editions of the race as well as constraints put on other similar sport events during this period, was restricted due to a relatively small sample size of participants manageable during the single day of investigation, which limits the precision of the estimate of the individuals affected by EIB during the four-minute competitions.

A high incidence of EIB was noted during the skyscraper stair race that was negatively associated with exercise performance. Therefore, EIB deserves more attention among active population undertaking intense exercise training and may require more careful management [14,17,18] to reduce the symptoms, improve fitness, and ensure continued sports and/or active leisure time participation of the affected individuals.

## Conclusions

In conclusion, we found a high incidence of EIB during a skyscraper (tower) stair race that was negatively associated with exercise performance. Thus, among the active population undertaking intense exercise training, EIB deserves more attention and may require more careful management to reduce the symptoms, improve fitness, and ensure continued sports and/or active leisure time participation of the affected individuals.
